# Oncogenic activity of SOX1 in glioblastoma

**DOI:** 10.1038/srep46575

**Published:** 2017-04-20

**Authors:** Idoia Garcia, Juncal Aldaregia, Jelena Marjanovic Vicentic, Paula Aldaz, Leire Moreno-Cugnon, Sergio Torres-Bayona, Estefania Carrasco-Garcia, Laura Garros-Regulez, Larraitz Egaña, Angel Rubio, Steven Pollard, Milena Stevanovic, Nicolas Sampron, Ander Matheu

**Affiliations:** 1 Cellular Oncology group, Biodonostia Health Research Institute, San Sebastian, Spain; 2 IKERBASQUE, Basque Foundation for Science, Bilbao, Spain; 3 CIBER de Fragilidad y Envejecimiento Saludable (CIBERfes), Madrid, Spain; 4 Laboratory for Human Molecular Genetics, Institute of Molecular Genetics and Genetic Engineering, University of Belgrade, Belgrade, Serbia; 5 Neuro-oncology Tumor Board, Donostia Hospital, San Sebastian, Spain; 6 Group of Bioinformatics, CEIT and TECNUN, University of Navarra, San Sebastian, Spain; 7 MRC Centre for Regenerative Medicine, University of Edinburgh, Edinburgh, UK

## Abstract

Glioblastoma remains the most common and deadliest type of brain tumor and contains a population of self-renewing, highly tumorigenic glioma stem cells (GSCs), which contributes to tumor initiation and treatment resistance. Developmental programs participating in tissue development and homeostasis re-emerge in GSCs, supporting the development and progression of glioblastoma. SOX1 plays an important role in neural development and neural progenitor pool maintenance. Its impact on glioblastoma remains largely unknown. In this study, we have found that high levels of *SOX1* observed in a subset of patients correlate with lower overall survival. At the cellular level, *SOX1* expression is elevated in patient-derived GSCs and it is also higher in oncosphere culture compared to differentiation conditions in conventional glioblastoma cell lines. Moreover, genetic inhibition of *SOX1* in patient-derived GSCs and conventional cell lines decreases self-renewal and proliferative capacity *in vitro* and tumor initiation and growth *in vivo*. Contrarily, SOX1 over-expression moderately promotes self-renewal and proliferation in GSCs. These functions seem to be independent of its activity as Wnt/β-catenin signaling regulator. In summary, these results identify a functional role for SOX1 in regulating glioma cell heterogeneity and plasticity, and suggest SOX1 as a potential target in the GSC population in glioblastoma.

Glioblastoma is the most common, aggressive and malignant adult brain tumor with an associated median survival of 15 months^1^. In recent years, several studies have provided a high-resolution picture of the genetic, epigenetic, and transcriptomic landscape of glioblastoma, revealing a large number of genetic mutations and molecular alterations that drive disease pathogenesis and establishing this type of tumor into biologically and clinically distinct subgroups^2^,^3^. Notably, the expression of the identified subtype classifiers varies across individual cells within a tumor, indicating significant intratumoral heterogeneity in glioblastoma^4^. This heterogeneity is a major challenge for targeted therapy.

Increasing evidence indicates that several transcription factors directing developmental decisions can also function as oncogenes by promoting the reacquisition of developmental programs required for tumorigenesis^5^. Moreover, certain malignant tumors depend on a cellular hierarchy, with privileged subpopulations, called cancer stem cells (CSCs), driving tumor spread and growth. Thus, developmental programs, which participate in tissue development and repair regulating normal stem and progenitor cells, re-emerge in CSCs to support the development and progressive growth of tumors^5^. Significant advances have been made in the identification of the molecular mechanisms underlying the pathobiology and intratumoral heterogeneity of glioblastoma^4^,^6^, however, further elucidation of the developmental programs governing glioma stem cells (GSCs) and glioblastoma progression is required in order to accelerate the development of urgently needed novel therapeutic targets and treatments.

*SOX* (sex-determining region Y (SRY)-box) genes are a family of transcription factors characterized by containing a conserved high-mobility group (HMG) DNA-binding domain. There are 20 members in humans divided into 8 groups based on their HMG sequence identity^7^. Members within the same group may have overlapping expression patterns, share biochemical properties, and perform synergistic or distinct functions. SOX family members play crucial roles in both embryonic and postnatal development. They are also important for stem cell regulation and maintenance, particularly in the central nervous system^8^,^9^. There is a growing body of evidence that several *SOX* members are involved in cancer development. In general, they play a role in tumors arising in tissues overlapping with their expression pattern during embryonic development. Notably, some members of the family are oncogenes while others act as tumor suppressors^10^. For example, SOX2, SOX4, SOX9 or SOX10 display oncogenic functions in different types of cancers including glioblastoma^11^,^12^,^13^,^14^,^15^,^16^. In contrast, SOX17 and SOX11 have been shown to act as tumor suppressors in certain types of cancer such as gastrointestinal tumors, mantle cell lymphomas, and also glioblastomas^17^,^18^,^19^,^20^,^21^.

SOX1 is a member of the SOXB1 subgroup, also containing SOX2 and SOX3. It is a well-established tumor suppressor in ovarian, hepatocellular, cervical and nasopharyngeal cancers whose expression is commonly silenced by hypermethylation of its promoter region^22^,^23^,^24^,^25^. These findings are in agreement with the notion that promoter hypermethylation of tumor suppressor genes is an important contributor to carcinogenesis^26^. Mechanistically, SOX1 functions as a tumor suppressor through interaction with β-catenin, and consequent inhibition of the Wnt signaling pathway^23^,^25^. During development, members of the SOXB1 subgroup, show distinct and overlapping expression patterns. Sox1 is the earliest known specific marker of the neuroectoderm lineage, being activated during gastrulation. The other members, Sox2 and Sox3, show broader expression patterns turning on at the pre-implantation and epiblast stages, respectively^27^,^28^. In the brain, several reports have shown that *Sox1* is a key regulator of neural progenitor identity and neural cell fate determination, maintaining the ability of these cells to either proliferate or differentiate from early development to adult stages^28^,^29^,^30^,^31^. Moreover, SOXB1 group members are coexpressed in the neural stem cell population and show certain degree of functional redundancy^8^,^32^. Regarding the activity of SOXB1 members in glioblastoma, the oncogenic function and clinical relevance of SOX2 is well established, most of its roles being linked to GSC regulation^14^,^33^,^34^,^35^,^36^. In contrast, little is known about the expression or function of SOX1 and SOX3. Interestingly, microarray analysis in *SOX2* knockdown glioma cells identified *SOX1* and *SOX18* among the almost 500 genes whose expression was altered^37^, and this allowed us to hypothesize that SOX1 might have a role in glioblastoma. In this study, we explore this hypothesis finding that *SOX1* is overexpressed in a subset of glioblastomas which present overall shorter patient survival. Moreover, we reveal that *SOX1* is highly enriched in the pool of GSCs and its inactivation significantly impairs their malignant properties.

## Material and Methods

### Patients and tumor samples

Human glioblastoma samples were provided by the Basque Biobank for Research-OEHUN (http://www.biobancovasco.org). Data for GBM and LGG was downloaded using TCGA-Assembler. The methods and experimental protocols in human samples were carried out in accordance with relevant guidelines, and all study participants signed the informed consent form. The study was approved by the ethic committee of Biodonostia Institute and Hospital Donostia.

### Cell culture

Glioma cell lines T98, A172, U87, U373 and U251 were obtained from the ATCC. Cells were cultured in DMEM (Gibco), supplemented with 10% fetal bovine serum (FBS), L-glutamine, penicillin and streptomycin (Gibco). Patient-derived GNS166 and GNS179 cell lines, kindly provided by Dr. Steven Pollard^38^, GB1 and GB2, established by our group^14^, and oncospheres derived from cell lines were cultured in DMEM/F12 (Gibco) supplemented with N2 and B27 (Fisher), 40% glucose (Sigma), and growth factors b-FGF2, and EGF (Sigma). Differentiation assays were performed by removing bFGF and EGF and by adding 1%FBS to the DMEM-F12 complete medium. To perform the spheres assay, 5 × 10^3^ cells were plated per triplicate and grown in DMEM/F12 complete medium for 10 days.

### Viral infections

Lentiviral infection was performed as described previously using a multiplicity of infection of 10 for 6 h^39^. For this, pLM-mCitrine-*SOX2* (SOX2) was received as a gift from Dr. Izeta^40^, pWPXL-SOX1 (SOX1) was cloned by Dr. Stevanovic, and pLKO.1 sh*SOX1 (sh1* and *sh5*) were obtained from Sigma. Cells transduced with pLKO.1 and shRNA plasmids were selected with 2 μg/ml puromycin (Sigma) and maintained with 0.2 μg/ml puromycin.

### Immunofluorescence

Immunofluorescence was performed following standard procedures^14^. The primary and secondary antibodies used were anti-phospho-histone H3 (p-H3, 1:2000; Ab14955, Abcam), β-catenin (1:250; 610153, BD transduction laboratories), anti-mouse Alexa Fluor 555 IgG (1:500 Invitrogen) and Cy3-streptavidin (1:5000, Jackson ImmunoResearch). Nuclear DNA staining and the mounting were performed with the Vectashield Hard set Mounting Medium with DAPI counterstain (Vector Laboratories). Pictures were taken in an Eclipse 80i microscope and processed with the NIS Elements Advanced Research Software (Nikon).

### Cell viability MTT assay

2 × 10^3^ cells per well were seeded in sextuplicate and after 24 h, 0.5 mg/ml Thiazolyl Blue Tetrazolium Bromide (MTT, Sigma) was added for 3 h at 37 °C. After the incubation, the content of the wells was removed and 150 μl DMSO were added in order to dilute the formazan salt formed by viable cells. Absorbance was measured at 570 nm in a MultiSkan Ascent microplate reader (Thermo Scientific) using the Ascent software. Cellular viability of the *shSOX1* cells was calculated relative to the absorbance of control cells.

### RNA extraction, reverse transcription and gene expression

Total RNA was extracted using Tri Reagent solution (Life Technologies). Reverse transcription was performed using random primers and the MultiScribe^TM^ Reverse Transcriptase Kit (Life Technologies). For qRT-PCR, 20 ng of cDNA was used to analyze gene expression with Absolute SYBR Green mix (Applied Biosystem), in a LightCycler 96 thermo-cycler (Roche). Transcript levels were normalized to *GAPDH* and measured using the ΔΔCt relative quantification method.

### Immunohistochemistry

Tumors generated in mice were collected, fixed in 10% formalin for 48 h and embedded in paraffin. 4 μm thick sections were deparaffinized, rehydrated and heated for 10 minutes in citrate buffer for antigen retrieval. Endogenous peroxidase was blocked with 5% hydrogen peroxide in methanol for 15 min. After incubation with blocking solution, sections were incubated with the respective primary antibody anti-Ki67 (AB15580, Abcam), SOX1 (4194, Cell Signaling), SOX2 (AB5603, Millipore) and PML (A301–167A, Bethyl Laboratories) at 37 °C for 2 hour. The sections were then washed and incubated with MACH 3 Rabbit Probe and MACH 3 Rabbit HRP-Polymer (M3R531, Biocare Medical). Color was developed with 3,3′ Diaminobenzidine (DAB) and nuclei were counterstained with hematoxylin.

### Western blot

Immunoblots were performed following standard procedures. The antibodies used in this study were anti-SOX1 (4194, Cell Signaling) and anti-SOX2 (AB5603, Millipore). Detection was performed by chemiluminescence using NOVEX ECL HRP Chemiluminescent Substrate Reagent Kit (WP20005, Invitrogen).

### *In vivo* carcinogenesis

All animal handling and protocols were approved by the animal care ethic committee of Biodonostia Institute. For xenotransplantation, GSCs were injected stereotactically into the frontal cortex of 6 to 8 week-old NOD-SCID mice. Briefly, GSCs were disaggregated with accutase and resuspended in PBS. Approximately 1 × 10^5^ cells were injected into the putamen using a stereotaxic procedure. Kaplan-Meier survival analysis was performed using the GraphPad Prism 5 software. For subcutaneous injection, glioma cells were harvested with trypsin/EDTA and resuspended in PBS. Approximately 5 × 10^5^ and 5 × 10^4^ cells were injected subcutaneously into both flanks of 8 week-old Foxn1^nu^/Foxn1^nu^ nude mice. Mice were observed on a weekly basis and external calipers were used to measure tumor size, and from these measurements, tumor volume was estimated by V = L*W^2^*0.5; where L is the tumor length and W is the tumor width.

### Data analysis

Results are represented as mean values ± standard error (SEM), indicating the number of experiments carried out for each assay. Statistical significance has been calculated using Student’s t-test, (*p ≤ 0.05; **p ≤ 0.01; and ***p ≤ 0.001), or the log-rank test for Kaplan Meier survival analyses.

## Results

### High *SOX1* expression is associated with poor clinical outcome in glioblastoma

**Figure 1:**
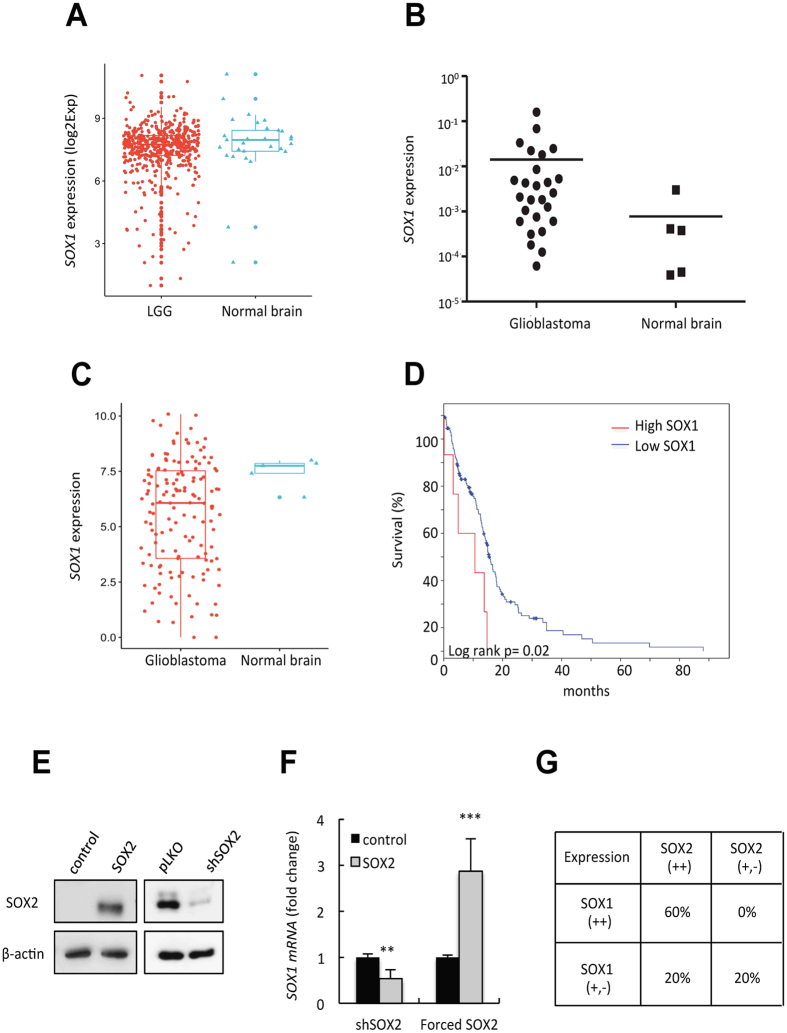
High levels of SOX1 are associated to poor clinical outcome and correlate with SOX2. (**A**) Boxplot of the log2 of the FPKM of LGG (low grade glioma) vs normal brain samples in TGCA. Wilcoxon test, p value = 0.17. (**B**) *SOX1* mRNA expression levels in GBM samples from Hospital Donostia and normal brain samples. (**C**) Boxplot of the log2 of the FPKM of glioblastoma vs normal brain samples in TGCA. The number of available RNAseq samples for glioblastoma is smaller than for LGG. Wilcoxon test, p value = 0.068. (**D**) Kaplan–Meier curves for the TCGA patient overall survival rates based on *SOX1* expression obtained from cbioportal. LogRank Test p = 0.02. (**E**) SOX2 protein expression in U87 cells transduced with ectopic *SOX2* and U251 cells infected with *shSOX2* and (**F**) *SOX1* mRNA levels in the indicated cells. qRT-PCR data are normalized to *GAPDH* expression and are expressed relative to the control condition (n ≥ 3). (**G**) Analysis of the correlation of *SOX2* and *SOX1* expression in human glioblastoma samples. Fisher exact test p < 0.05.

We analyzed the expression of *SOX1* transcription factor in human clinical biopsies from brain tumors. First, we compared the expression of *SOX1* in human low-grade glioma and normal brain samples. There were no differences between these two groups in The Cancer Genome Atlas (TCGA)^6^ publicly available datasets (Fig. 1A). Next, we investigated *SOX1* levels in a small glioblastoma cohort derived from Donostia Hospital. The expression of *SOX1* in the tumor biopsies varied between 0.12 and 133 fold change when compared to normal brain tissue, with 18 of 26 tumors showing greater than 1.5 fold change in *SOX1* levels (Fig. 1B). We also studied *SOX1* expression in the GBM data from the TCGA and found that its levels were also heterogeneous within the different samples (Fig. 1C). Notably, when we explored the correlation of *SOX1* levels with clinical characteristics in the TCGA cohort, high *SOX1* expression was associated with shorter overall survival (p = 0.02; Fig. 1D). Together, these results show that *SOX1* expression is elevated in a subset of glioblastoma samples and its expression is a prognostic biomarker.

### SOX2 regulates the expression of SOX1 in glioblastoma

Since transcriptomic studies found *SOX1* within the list of genes down-regulated in *SOX2-*silenced LN229 glioma cells^37^, and we have recently observed that SOX2 activity modulates proliferation and self-renewal in glioma cells^14^, we investigated whether the expression of SOX1 was regulated by SOX2 in glioma. Notably, we found that *SOX2*-silenced U251 cells (with high endogenous SOX2 levels) displayed lower *SOX1* levels than control cells (Fig. 1E,F). Contrarily, ectopic *SOX2* overexpression in U87 cells (with low endogenous SOX2) significantly increased the expression of *SOX1* (Fig. 1E,F). To further study this putative correlation between SOX2 and SOX1, we moved to clinical biopsies, analyzing the expression of those transcription factors in the Donostia Hospital cohort of human glioblastoma samples. Interestingly, the correlation analysis showed a significant association between *SOX2* and *SOX1* expression (Fig. 1G). In fact, 60% of the biopsies with *SOX2* overexpression also presented elevated levels of *SOX1*, whilst all of those with moderate or low *SOX2* also had low *SOX1* levels. These results indicate that there is a positive relationship between SOX2 and SOX1, it being likely that they act in the same signaling pathway.

### GSCs express high levels of SOX1

**Figure 2:**
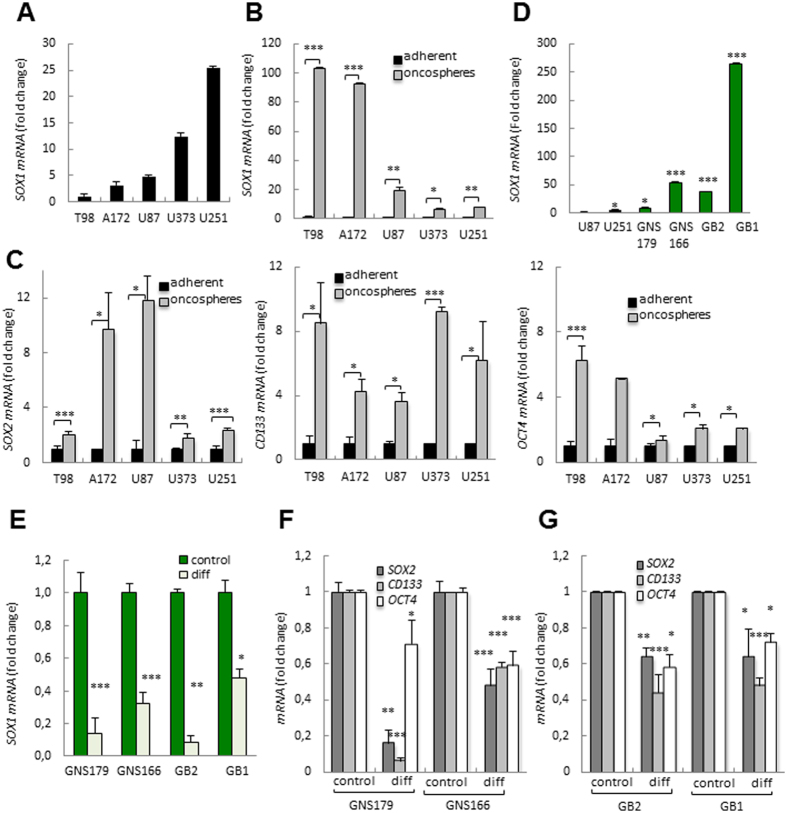
*SOX1* enrichment in glioma stem cell populations. (**A**) *SOX1* mRNA levels in the indicated glioma cell lines showing different expression levels among them (n ≥ 3). (**B**) *SOX1* levels in each cell line grown in stem cell medium (oncospheres) relative to in serum (adherent) (n ≥ 2). (**C**) mRNA levels of the indicated stem cell markers grown in serum and stem cell medium (n ≥ 2). (**D**) *SOX1* expression levels in U87 and U251 conventional cell lines and 4 patient derived GSC lines, the expression is relative to U87 cell line (n ≥ 3). (**E**) *SOX1* levels in four GSC lines grown in stem cell medium (control) compared to differentiation conditions (diff) (n ≥ 2). (**F**,**G**) mRNA levels of the indicated stem cell markers grown in stem cell medium (control) compared to differentiation conditions (diff) in GNS and GB cells respectively (n ≥ 2). qRT-PCR data are normalized to GAPDH expression.

We cultured several conventional glioma cell lines in different conditions, growing them as adherent monolayers in the presence of serum (adherent) and as oncospheres in stem cell media. We first analyzed the expression levels of *SOX1* in the adherent cells finding high levels in U251 and U373 cells, and low levels in U87, A172 and T98 cells (Fig. 2A). Interestingly, oncospheres derived from all five glioma cell lines had higher levels of *SOX1* than observed in adherent cells (Fig. 2B). The latter culture condition was accompanied by increased expression of stem cell markers (*SOX2, CD133*, and *OCT4*) (Fig. 2C), which is consistent with an enrichment of stemness activity. Notably, we have previously shown that oncospheres, in line with their enhanced tumor-propagating activity, were associated with the formation of larger and faster-growing tumors^14^.

To further characterize the expression of SOX1, we moved onto primary GSCs derived from human patients, a model that is more similar and hence relevant to the clinical situation. We studied the levels of SOX1 expression in four independent GSC cultures detecting markedly higher levels in GSCs than the conventional glioma cell lines (Fig. 2D). Next, we investigated the relationship between *SOX1* and the population of GSCs after differentiation of the four GSC primary cultures by removing the EGF and b-FGF2 growth factors and by adding serum. In this context, the levels of *SOX1* decreased dramatically, by a mean of 70%, in all four cases (Fig. 2E). Similar results were observed in *SOX2, CD133* and *OCT4* stem cell markers (Fig. 2F,G). These results demonstrate that SOX1 levels are highly enriched in GSCs and correlate with the glioma cell undifferentiated condition.

### *SOX1* knockdown inhibits GSC activity

**Figure 3:**
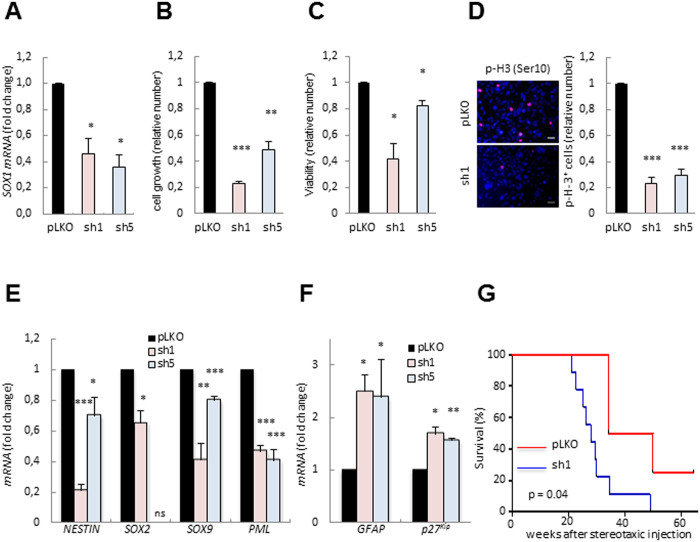
SOX1 knockdown impairs self-renewal and tumor growth in GSCs. (**A**) *SOX1* mRNA expression in control (*pLKO*) and sh*SOX1 (sh1* and *sh5*) GNS166 (n ≥ 2). (**B**) Cell growth at day 5 comparing *pLKO* and *shSOX1* GNS166 cells (n = 3). (**C**) MTT studies measuring cell viability in *shSOX1* relative to control GNS166 cells (n = 3). (**D**) Representative image and quantification of number of p-H3 positive cells in *pLKO* and *shSOX1* transduced GNS166 cells (n = 3). (**E**) mRNA levels of the indicated stem cell markers in *sh1* and *sh5* GNS166 cells relative to control expression (n ≥ 2). (**F**) *GFAP* and *p27*^*Kip*^ mRNA levels in the indicated cell conditions (n ≥ 2). (**G**) Kaplan-Meier curve representing the survival of NOD-SCID mice that were xenotransplantated with control (n = 9) and *sh1* (n = 4) GNS 166 cells.

To directly explore the role of SOX1 in the activity of GSCs, we knocked-down *SOX1* expression in a patient-derived cell line (GNS166) with two independent shRNAs. Effective inhibition of *SOX1* was demonstrated with qRT-PCR when using both sh*SOX1* constructs (*sh1* and *sh5*) (Fig. 3A). Functionally, *SOX1* silencing promoted a significant decrease of more than 2-fold in cell growth rates (Fig. 3B). In line with this, MTT studies showed that the cell viability rate was also diminished in *SOX1* silenced GNS166 cells (Fig. 3C). These phenotypes correlated with a reduction in the number of the proliferative marker p-H3 positive cells (Fig. 3D). Specifically, we detected decrease in more than 70% of proliferating cells in *sh1* and *sh5* than in empty vector cells (Fig. 3D).

To further determine the impact of SOX1 regulating GSC self-renewal, we measured the expression of several stem cell and differentiation markers. Notably, we observed a reduction in *NESTIN, SOX2, SOX9* and *PML* stem cell markers (Fig. 3E), concomitantly with an increase in the expression levels of *GFAP* and *p27*^*Kip*^ (Fig. 3F). Taken together, these results show that SOX1 plays a relevant role in GSC plasticity, via the regulation of the stemness-differentiation dichotomy.

The gold standard to identify the presence of GSC is to analyze the ability of the original patient tumor to replicate the tumor formation ability *in vivo* when transplanted orthotopically^41^. Therefore, NOD-SCID mice were intracranially injected with *pLKO* and *sh1* GNS166 cells. Interestingly, *SOX1* silencing significantly delayed tumor-forming capacity of GNS166 cells (Fig. 3G). Thus, the median survival for mice injected with *pLKO* cells was 27 weeks, whereas mice injected with *sh1* cells survived a median of 42 weeks. These results indicate that SOX1 regulates GSCs self-renewal and tumorigenic activity.

### *SOX1* knockdown inhibits tumor initiation and progression in U251 glioma cells

**Figure 4:**
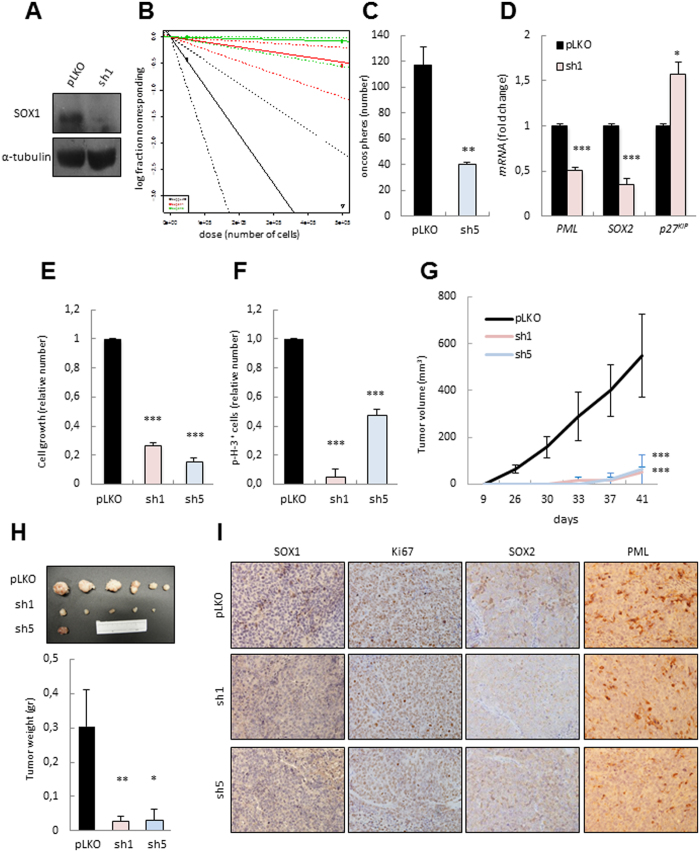
SOX1 knockdown in the U251 glioma cell line decreases tumor initiation and progression. (**A**) Representative western blotting of SOX1 protein expression in U251 cells infected with p*LKO* or *sh1* (n = 3). (**B**) Frequency of tumor initiation after subcutaneous injection in nude mice of 5 × 10^5^ and 5 × 10^4^ U251 cells transduced with *pLKO, sh1* or *sh5*. The incidence of tumor initiation was measured using the ELDA platform. (**C**) Quantification of the number of spheres formed from the indicated conditions (n = 3). (**D**) mRNA levels of the indicated genes in *sh1* U251 cells relative to empty vector (n = 3). (**E**) Cell growth of U251 cells transduced with *sh1* and *sh5* relative to *pLKO* cells (n = 3). (**F**) Quantification of the number of p-H3 positive cells in the same conditions (n = 3). (**G**) Volume of tumors generated after subcutaneous injection of U251 p*LKO, sh1* or *sh5* cells (n = 12) at the indicated time-points. (**H**) Picture and average weight of the tumors generated in (**G**). (**I**) Representative images of the immunohistochemical staining of KI67, SOX1, SOX2 and PML in tumors from G (n = 4).

In order to determine whether the mechanism by which SOX1 regulates proliferation and tumor growth is specific to GSCs or it is broader, we knocked-down *SOX1* expression in the U251 cell line. Western blotting demonstrated effective inhibition of SOX1 at protein levels (Fig. 4A). Tumor-initiating ability in limiting dilution and oncosphere formation studies functionally defines self-renewing CSCs *in vivo* and *in vitro* (Clevers CSCs premises). Therefore, we tested whether SOX1 silencing could regulate tumor initiation performing subcutaneous inoculation of serial diluted U251 cells transduced with empty vector or both sh*SOX1* constructs (*sh1* and *sh5*) in immunodeficient mice and by performing oncosphere formation assays. Strikingly, the frequency of tumor initiating was 1/1050263 in *sh1* and 1/6359439 *sh5* cells compared to 1/108183 in the empty vector harbouring cells (Fig. 4B). In line with these results, SOX1 silencing markedly decreased the ability of U251 cells to generate oncospheres (Fig. 4C). Moreover, at the molecular level, the silencing of *SOX1* decreased *PML* and *SOX2* expression (Fig. 4D), but up-regulated *p27*^*Kip*^ levels (Fig. 4D). These results phenocopy the data obtained in GSCs and further reinforce that SOX1 silencing display a robust effect on blocking self-renewal and tumor initiation.

We further evaluated the role of *SOX1* silencing in glioma cells. At the cellular level, cell counting studies revealed a significant reduction of 70% in cell growth rates in *SOX1*-silenced U251 cells (Fig. 4E). Moreover, the number of p-H3 positive cells was reduced by a mean of 90% and 50% in the case of *sh1* and *sh5* respectively, indicating that cell proliferation was dramatically impaired when *SOX1* is down-regulated (Fig. 4F). Furthermore, there was a significant decrease in tumor growth in *shSOX1* cells (Fig. 4G,H). Indeed, *sh1* and *sh5* cells formed subcutaneous tumors reaching less than 75 mm^3^ 40 days after injection, while control tumors grew to an average of 550 mm^3^ in the same period of time (Fig. 4G). The impaired tumorigenic ability of *shSOX1* cells was further corroborated by immunohistochemistry analysis in the tumors *in vivo*. Indeed, *sh1* and *sh5* derived xenografts possessed lower number of SOX1, Ki67, SOX2 and PML positive cells than tumors derived from control cells (Fig. 4I). In summary, SOX1 genetic silencing induces a strong tumor suppressor phenotype in glioma cells associated with impaired self-renewal, proliferation, tumor initiation and progression.

### SOX1 activity is not mediated by *WNT/β-catenin* signaling pathway

**Figure 5:**
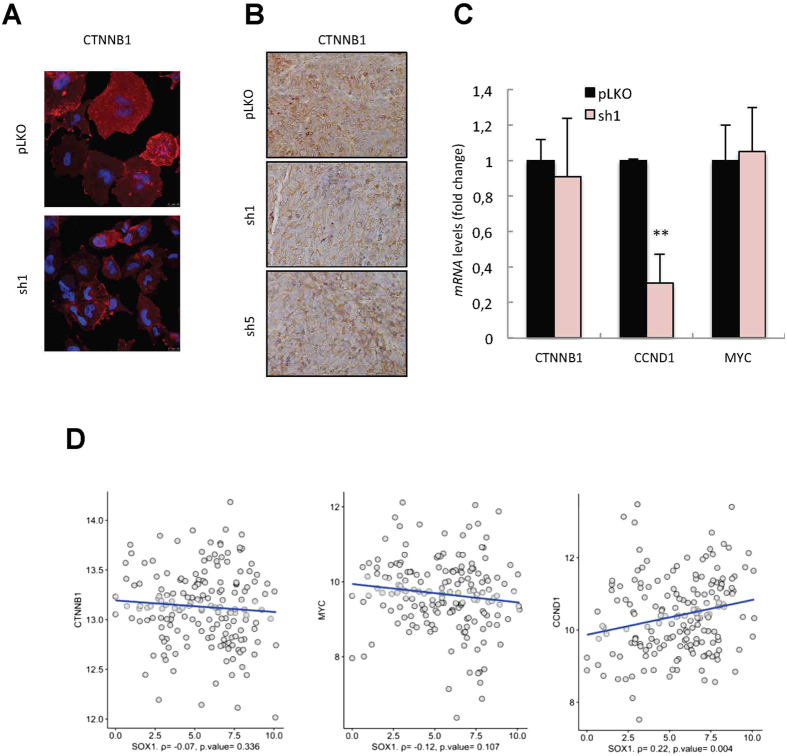
SOX1 is not regulating the WNT signaling pathway in glioblastoma. (**A**) Representative images of CTNNB1 (β-catenin) immunofluorescence staining in U251 plko and *sh1* cells (n = 4). (**B**) Representative images of CTNNB1 immunohistochemical staining in U251 p*LKO, sh1* and *sh5* derived tumors (n = 4). (**C**) mRNA levels of *CTNNB1, CCND1* (CYCLIN D1) and *MYC* in GNS166 p*LKO* and *sh1* cells. qRT-PCR data are normalized to *GAPDH* expression (n ≥ 2). (**D**) Scatter plot of log2 of the FPKM of *CTNNB1, MYC* and *CCND1* vs *SOX1* expression. In the x-axis, the correlation and its statistical significance are included. Only *CCND1* has a significant correlation with *SOX1*.

Since SOX1 acts as a tumor suppressor in different types of cancer through the *Wnt/β-catenin* signaling pathway (see introduction), we examined the activity of this pathway after silencing of *SOX1* in glioma cells and GSCs. Immunofluorescence and immunohistochemistry of β-catenin did not show any clear difference in its expression and nuclear translocation between U251 cells transduced with empty vector or sh*SOX1* constructs (Fig. 5A,B). Moreover, qRT-PCR analysis in *SOX1*-silenced GNS166 cells did not show any significant modification in *β-catenin* and *MYC* expression levels (Fig. 5C), the latter being a well-established *β-catenin* downstream target^39^. To pursue the association between SOX1 and β-catenin, we turned into human patient biopsies. The results at cellular level were further confirmed in the datasets of TCGA cohort, where correlation analysis did not find association between *SOX1* and *β-catenin* or *MYC* expression levels (Fig. 5D). These results suggest that the oncogenic activity of *SOX1* is not mediated in glioblastoma cells by the β-catenin signaling pathway both at cellular level and in clinical samples.

We also studied the expression of *CYCLIN D1,* an additional *β-catenin* downstream target^39^. In this case, *shSOX1* GNS166 cells presented diminished levels of *CYCLIN D1* (Fig. 5C), and interestingly its expression significantly correlated to *SOX1* in the TCGA datasets (p < 0.005) (Fig. 5D). These results postulate *CYCLIN D1* as a putative mediator of SOX1 activity in glioblastoma.

### Ectopic SOX1 overexpression promotes GSC proliferation and self-renewal

**Figure 6:**
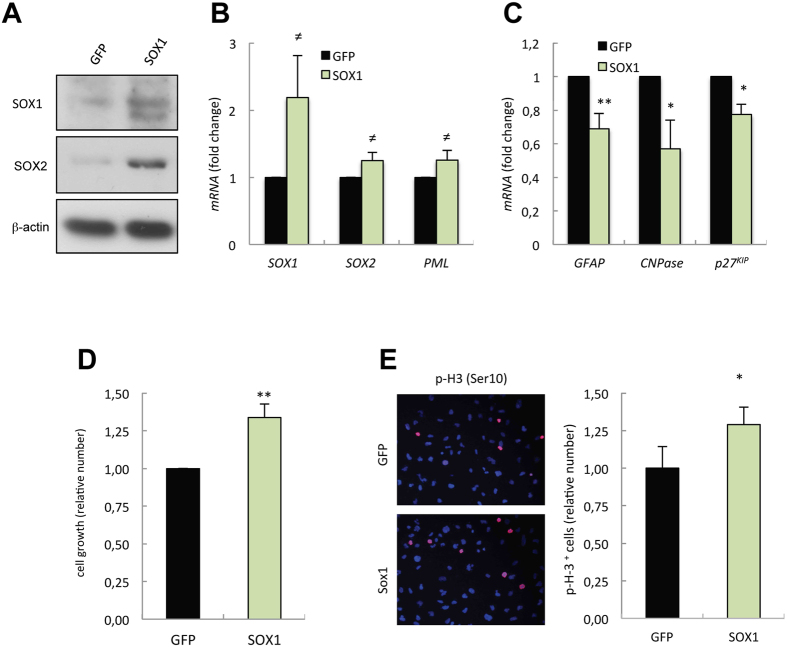
SOX1 overexpression contributes to the malignant phenotype in GSCs. (**A**) SOX1 and SOX2 protein expression in GNS166 cells transduced with ectopic *SOX1* (n = 3). (**B**) mRNA levels of the indicated stem cell markers in GNS166 cells transduced with SOX1 relative to control (GFP) expression (n ≥ 3). (**C**) mRNA levels of the indicated differentiation markers in the same cells (n ≥ 3). (**D**) Cell growth at day 5 comparing control (GFP) and *SOX1* overexpressing GNS166 cells (n = 6). (**E**) Representative image and quantification of the number of p-H3 positive cells in *SOX1* transduced GNS166 cells compared to control (GFP) condition (n = 6).

Finally, we introduced a construct encoding *SOX1* gene sequence in GNS166 cells. We confirmed the overexpression of SOX1 by Western blotting and q-RT PCR (Fig. 6A,B). In this context, *SOX1* overexpression slightly increased the expression of *SOX2* and *PML* stem cell markers (Fig. 6A,B), whilst decreased *GFAP, CNPase* and *p27*^*Kip*^ levels (Fig. 6C). Phenotypically, cells with increased *SOX1* expression exhibited moderately higher cell growth curves (Fig. 6D), and rates of proliferation compared to control cells (Fig. 6E). Collectively, this data revealed that elevated activity of SOX1 is not only necessary for the maintenance but might also promote proliferative and self-renewal activity in GSCs.

## Discussion

Several transcription factors that direct developmental decisions might also act as oncogenes by promoting reactivation of programs required for tumorigenesis^5^. SOX1 is a transcription factor that is essential for maintaining proliferation in the neural stem/progenitor pool, but its continued expression, leads to neuronal differentiation during development and adult stages^42^. Loss of SOX1 leads to epilepsy and eventual death though its absence is partially compensated for the other members of the SOXB1 subgroup, SOX2 and SOX3, with which shows overlapping expression patterns in neural stem/progenitor cells, and counteracts the activity of proneural proteins^28^,^32^,^43^,^44^. Based on this evidence, SOX1 might be considered a key player in neural development through the maintenance of neural/progenitor pool homeostasis. Prior to this study, little was known about the impact of SOX1 in glioblastoma and in the maintenance of the GSC population. In this work, we have identified that SOX1 displays oncogenic activity in glioblastoma by using several different approaches.

First, we investigated the expression of *SOX1* in human brain samples. The analysis of *SOX1* expression at mRNA level in a cohort of glioblastoma patients from Donostia Hospital indicated that *SOX1* expression was slightly up-regulated in around 60% of tumor tissues compared to levels in healthy human brain samples. Taking advantage of the publicly available TCGA cohort data, we found that high levels of *SOX1* in a subgroup of patients were associated with shorter patient survival. These data confirm the clinic-pathological and prognostic significance of *SOX1* expression, and, to our knowledge, it is the first evidence of high SOX1 expression level as a negative prognostic biomarker in cancer. In fact, low expression of SOX1 protein and/or mRNA expression was correlated with shorter overall survival and poor prognosis in ovarian cancer^22^, human hepatocellular carcinoma^23^,^45^, and esophageal squamous cell carcinoma^46^,^47^. These two sets of observations are not conflicting *per se* because it is conceivable that the expression of SOX1 could be elevated or decreased depending on the epigenetic status or the cellular heterogeneity and plasticity. Regarding the epigenetic status, lower levels of SOX1 and better prognostic significance has been linked to the methylation of its promoter in several types of cancer^23^,^25^,^46^. Regarding cellular heterogeneity and plasticity, our data revealed that *SOX1* expression is enriched in the population of GSCs, grown in stem cell media, compared to parental cells, cultured in the presence of serum. Moreover, we found that patient-derived GSCs have higher levels of *SOX1* expression than conventional cell lines, and these levels decrease when the GSCs are induced to differentiate in the presence of serum. These results demonstrate that high levels of *SOX1* are linked to maintaining GSCs in an undifferentiated state. In agreement with this idea, *SOX1* has been identified within the set of 19 neurodevelopmental transcription factors that are active and have higher expression in GSCs than in differentiated cells^48^. Furthermore, mapping of chromatin accessibility, before and after differentiation with BMP treatment, identified several enriched motifs for SOXB1 family members, mostly SOX2 but also SOX1, as regulatory regions that failed to be completely silenced in GSC settings^49^. Moreover, *SOX1* has been observed among the set of genes with elevated expression in CD44+/CD24− and CD133+ breast cancer stem cells^50^ and in invasive prostate cancer cells, where *SOX1* promoter was hypomethylated^51^. Together, these results postulate that the enrichment of *SOX1* in the population of CSCs is likely to be mediated by temporal and context dependent epigenetic changes. These findings are supported with the evidence that, during tumor initiation and progression, the epigenome of cancer cells goes through multiple alterations presenting broad domains of promoter hypermethylation, contributing to carcinogenesis through the inactivation of tumor suppressor genes and epigenetic regulators; but also including a genome-wide loss of DNA methylation (hypomethylation), likely affecting transcription factors which are important for self-renewal, and which are, therefore, under selective pressure to maintain or increase their expression in the corresponding cancer cell^26^,^49^,^52^.

Next, we studied the role of SOX1 in glioma cell activity through knockdown and overexpression assays. Experimental silencing of *SOX1* directly in GSCs markedly reduced their proliferative and self-renewal activity, and delayed the formation of tumors when the cells were xenotransplanted into the brain. When the same approach was used with U251 cells, we obtained similar results. Indeed, *SOX1* knockdown significantly impaired self-renewal and proliferative capability *in vitro* and tumor initiation and tumor progression *in vivo*. These results indicate that SOX1 expression is necessary for GSC maintenance likely regulating the interplay between proliferation, self-renewal and differentiation. On the contrary, *SOX1* overexpression in GSCs moderately increased cell growth, proliferation and expression of stem cell markers. A complementary study showed that elevated SOX1 in differentiated glioma cells modestly enhanced sphere formation, and weakly induced the expression of the stem cell marker CD133 but failed to initiate tumors in mice that received an orthotopic xenograft^48^. These results support the notion that elevated expression of SOX1 is essential for maintaining, but not sufficient for promoting the self-renewal of GSCs. Several additional factors might cooperate to activate stem cell-like properties. Indeed, POU3F2, SOX2, SALL2, and OLIG2 have been shown to be the core set of transcription factors essential for GBM propagation, which are within the set of 19 transcription factors (including SOX1) required for successful reprogramming of differentiated glioma cells into GSCs^48^. In summary, our results firmly establish that SOX1 behaves as an oncogene in glioblastoma regulating glioma cell plasticity. This activity contrasts the evidence available for other types of cancers such as hepatocellular or nasopharyngeal carcinoma^23^,^25^, cervical^24^, lung^53^, or breast cancers^54^, in which it displays tumor suppressor activity. These data underline the fact that the activity of SOX1 is context dependent in cancer.

It has been previously shown that SOX1 is a negative regulator of WNT/β-catenin signaling in several types of cancer justifying its tumor suppressor activity. In glioblastoma, however, we have not detected any remarkable effects of *SOX1* silencing on difference in the expression of β-catenin and its downstream target MYC at a cellular level *in vitro*, in tumors *in vivo*, as well as in clinical biopsies. Therefore, the oncogenic functions of SOX1 in glioblastoma seem to be β-catenin independent. Similar to these results SOX1overexpression in the embryonal teratocarcinoma cell line, NT2/D1, did not affect the activity of WNT signaling^55^. At the molecular level, we detected that gain and silencing of *SOX1* expression, in both GSC and U251 cell contexts, modulated the expression of the stem cell markers SOX2 and *PML*^56^,^57^,^58^, and well-established cell cycle regulators such as *p27*^*Kip*^ and *CYCLIN D1*^59^. These results suggest SOX2-PML and p27^Kip^-CYCLIN D1 as downstream molecular effectors by which SOX1 functions in glioblastoma governing self-renewal and proliferation programs. Additional studies have shown that SOX1 alters SOX2 expression in human laryngeal squamous cell carcinoma^60^, regulates p27^KIP^ levels in hepatocellular carcinoma^23^, and modulates CYCLIN D1 expression in hepatocellular and nasopharyngeal carcinoma as well as in breast cancer^23^,^25^,^54^. All together, data presented reinforce the relevance of those genes underlying SOX1 activity. However, further work is needed to define their interactions in glioblastoma.

In summary, our work has identified that *SOX1* expression is highly enriched in the pool of GSCs and its inactivation significantly impairs their malignant properties including proliferation, ability of self-renewal, differentiation capacity as well as tumor initiation and progression. Based on our results, we postulate that SOX1 is a master developmental transcription factor, governing and maintaining cellular plasticity and heterogeneity associated with diverse regulatory programs. Moreover, we reveal that *SOX1* is overexpressed in a subset of glioblastoma human biopsies and that its high levels are associated with shorter overall patient survival. Taken together, our data pointed out to a previously unappreciated role for SOX1 as a central player to glioblastoma biology, prognosis, and therapy.

## Additional Information

**How to cite this article:** Garcia, I. *et al*. Oncogenic activity of SOX1 in glioblastoma. *Sci. Rep.* **7**, 46575; doi: 10.1038/srep46575 (2017).

**Publisher's note:** Springer Nature remains neutral with regard to jurisdictional claims in published maps and institutional affiliations.
